# HIV-1 gp120 Induces Expression of IL-6 through a Nuclear Factor-Kappa B-Dependent Mechanism: Suppression by gp120 Specific Small Interfering RNA

**DOI:** 10.1371/journal.pone.0021261

**Published:** 2011-06-21

**Authors:** Ankit Shah, Ashish S. Verma, Kalpeshkumar H. Patel, Richard Noel, Vanessa Rivera-Amill, Peter S. Silverstein, Suman Chaudhary, Hari K. Bhat, Leonidas Stamatatos, Dhirendra P. Singh, Shilpa Buch, Anil Kumar

**Affiliations:** 1 Division of Pharmacology, School of Pharmacy, University of Missouri-Kansas City, Kansas City, Missouri, United States of America; 2 Ponce School of Medicine, Ponce, Puerto Rico; 3 Seattle Biomedical Research Institute, Seattle, Washington, United States of America; 4 University of Nebraska Medical Center, Omaha, Nebraska, United States of America; University of Rochester, United States of America

## Abstract

In addition to its role in virus entry, HIV-1 gp120 has also been implicated in HIV-associated neurocognitive disorders. However, the mechanism(s) responsible for gp120-mediated neuroinflammation remain undefined. In view of increased levels of IL-6 in HIV-positive individuals with neurological manifestations, we sought to address whether gp120 is involved in IL-6 over-expression in astrocytes. Transfection of a human astrocyte cell line with a plasmid encoding gp120 resulted in increased expression of IL-6 at the levels of mRNA and protein by 51.3±2.1 and 11.6±2.2 fold respectively; this effect of gp120 on IL-6 expression was also demonstrated using primary human fetal astrocytes. A similar effect on IL-6 expression was observed when primary astrocytes were treated with gp120 protein derived from different strains of X4 and R5 tropic HIV-1. The induction of IL-6 could be abrogated by use of gp120-specific siRNA. Furthermore, this study showed that the NF-κB pathway is involved in gp120-mediated IL-6 over-expression, as IKK-2 and IKKβ inhibitors inhibited IL-6 expression by 56.5% and 60.8%, respectively. These results were also confirmed through the use of NF-κB specific siRNA. We also showed that gp120 could increase the phosphorylation of IκBα. Furthermore, gp120 transfection in the SVGA cells increased translocation of NF-κB from cytoplasm to nucleus. These results demonstrate that HIV-1 gp120-mediated over-expression of IL-6 in astrocytes is one mechanism responsible for neuroinflammation in HIV-infected individuals and this is mediated by the NF-κB pathway.

## Introduction

Highly active anti-retroviral therapy has significantly reduced the incidence of HIV-associated dementia (HAD). However, HIV-associated neurocognitive disorders (HAND) remain a major problem in people infected with HIV-1. Although the pathogenic mechanisms responsible for HAND are uncertain, astrocytes are thought to play a major role in the disorder. Astrocytes are the most abundant cell type found in the neuroectodermal region and have been shown to be associated with various pathological abnormalities of the brain such as increased glutamate uptake, hypoxia, increased oxidative stress and disruption of blood-brain barrier integrity [1]. Astrogliosis has been reported in the brains of patients with HAD [2]. Astrocytes undergo activation in response to disorders in the CNS that involve injury and inflammation, including cerebral ischemia [3], multiple sclerosis [4], Alzheimer's disease [5], and human immunodeficiency virus type 1 encephalitis (HIVE) [6]. Li et al. showed that the intact HIV-1 virion can alter the expression of various cytokines in human fetal astrocytes [2].Viral proteins, such as Tat and gp120, have been implicated in pathways that involve direct as well as indirect toxicities to glial cells of the CNS, including astrocytes [7], [8], [9]. HIV-1 gp120 is a surface glycoprotein, which not only enables viral attachment and entry into the host cells, but has also been found to be involved in neurotoxicty [10], [11]. The mechanism of gp120-mediated neurotoxicity is known to involve oxidative stress [12], [13], [14] and induction of IL-1β production by glial cells [15]. Ronaldson et al. showed that gp120 plays a role in regulating transporter expression in rat astrocytes, presumably through the action of inflammatory mediators such as TNF-α, IL-1β, and IL-6 [16].

IL-6 is an activator of acute phase responses and is involved in crosstalk with other inflammatory mediators [17], [18]. IL-6-mediated inflammation is known to cause a higher incidence of gliosis and dendritic damage in patients with Parkinson's disease (PD), amyotrophic lateral sclerosis [10], multiple sclerosis [17] and Alzheimer Disease [19] [20], [21]. Furthermore, increased IL-6 and IL-8 levels have also been reported in HIV-1 infected patients, suggesting a possible link between cytokine levels and neuroAIDS [22]. Using mixed cultures of primary brain cells Yueng et al. demonstrated an increased expression of IL-6 in response to gp120 [23]. Another study by Kong et al. also demonstrated that gp120 could induce IL-6 in murine primary mixed glial cell cultures [24]. While cell culture models have demonstrated the induction of IL-6 along with other cytokines such as TNF-α and IL-1β a central role for IL-6 in gp120-induced neuroinflammation has been demonstrated using a rat model [25]. In this case, intrathecal administration of gp120 was shown to induce the expression of IL-6, TNF-α, and IL-1β. However, of critical importance is that treatment of the animals with antibody to IL-6 abrogated the expression of the other cytokines [25]. This suggests that IL-6 is capable of regulating other cytokines that are involved in mediating neuroinflammation.

Thus, determination of the mechanisms responsible for the gp120-mediated increase in IL-6 expression in astrocytes could provide information crucial for the treatment of neuroinflammation. To answer these questions we used a human astrocyte cell line, SVGA, to determine the effect of gp120 on IL-6 expression at the RNA and protein levels. Furthermore, these effects were confirmed in primary human fetal astrocytes by exposing these cells to gp120 protein. We also determined whether NF-κB inhibitors or siRNAs targeted towards either gp120 or NF-κB could block IL-6 upregulation by gp120.

## Results

### HIVgp120 induces a time-dependent IL-6 up-regulation

**Figure 1:**
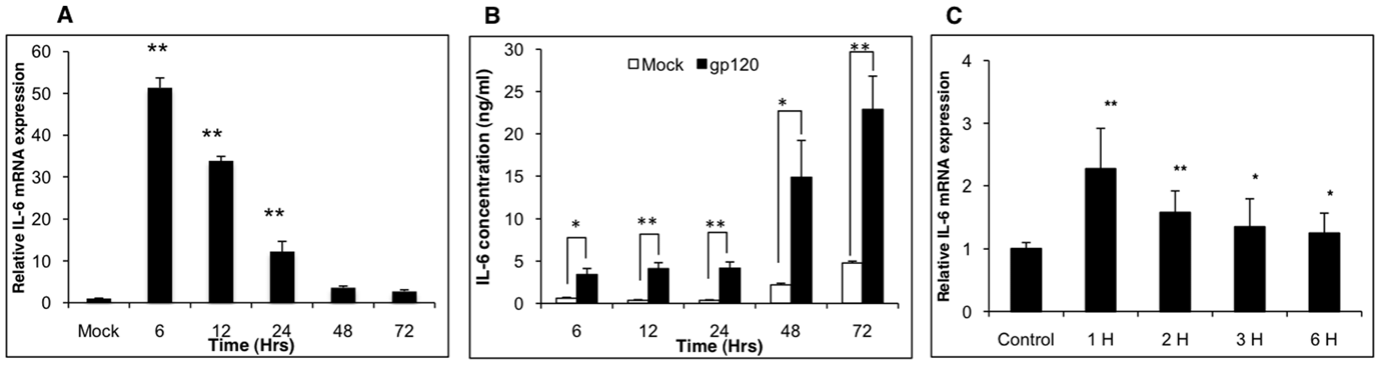
gp120-mediated increase of IL-6 expression in SVGA astrocyte cells. 1×10^6^ SVGA astrocytes were transfected with 1 µg gp120 JR-FL (R5 tropic) DNA using Lipofectamine™ 2000. The cells were harvested at the times described in the text. Mock transfection was performed with transfection of equal amount of empty human vector pcDNA3.1. All times noted on the figure and listed in the text are the times at which cells and supernatants were harvested after the end of the 5 hour transfection protocol. IL-6 mRNA (A) and IL-6 protein (B) expression levels were measured using real time RT-PCR and bioplex assays respectively. In (B), open bars show empty-vector transfected mock controls and closed bars show gp120 transfected samples. (C) shows the effect of exogenous gp120 on SVGA cells. SVGA were exposed to 20 nM recombinant gp120IIIB for various lengths of time and total mRNA was isolated. IL-6 expression levels were measured using real time RT-PCR. The mRNA levels are presented as fold difference between gp120 transfected cells and control cells transfected with empty plasmid. The protein concentration is presented as ng/ml protein in supernatant. Each bar represents mean ± SE of 3 experiments with each experiment done in triplicates. The statistical significance was calculated using student's t test and * and ** denotes p value of ≤0.05 and ≤0.01, respectively.

We first wanted to confirm that human astrocytes are a potential source of gp120-mediated IL-6 over-expression. SVGA cells were transfected with a gp120-expressing plasmid. Our transfection efficiency ranged between 55–75% as shown demonstrated by transfection of a plasmid encoding green fluorescent protein followed by flow cytometric analyses (data not shown). IL-6 mRNA was up-regulated and reached a peak level (51.3±2.1 fold) at 6 hours after transfection (Fig. 1A). IL-6 mRNA expression diminished from this peak level and was found to be 33.8±1.1, 12.3±2.4, 3.6±0.4 and 2.7±0.4 fold higher than in empty vector-transfected mock controls after 12, 24, 48 and 72 hour post-transfection, respectively (Figure 1A). We also quantified IL-6 protein concentrations at these time points. IL-6 protein was observed to be at significantly elevated levels as early as 6 hours post-transfection in supernatants of gp120-transfected cells compared to that in mock-transfected controls (3.418±0.708 vs 0.370±0.068 ng/ml) (Figure 1B). The IL-6 concentration increased in both control and gp120-transfected cells over time but gp120-transfected cells showed significantly elevated levels compared to the control. The IL-6 concentration was 4.8 to 11.6 fold-higher in gp120-transfected cells compared to those in control wells (Figure 1B). Previous studies have reported that astrocytes express the CXCR4 chemokine receptor [26], [27]. Therefore, gp120IIIB which can bind to CXCR4 was used in this study in order to determine the effect of exogenous gp120 upon astrocytes. SVGA cells were treated with 20 nM gp120IIIB. Increased levels of IL-6 expression were found as soon as 1 hour, and these levels gradually declined over the next 5 hours (Figure 1C).

**Figure 2:**
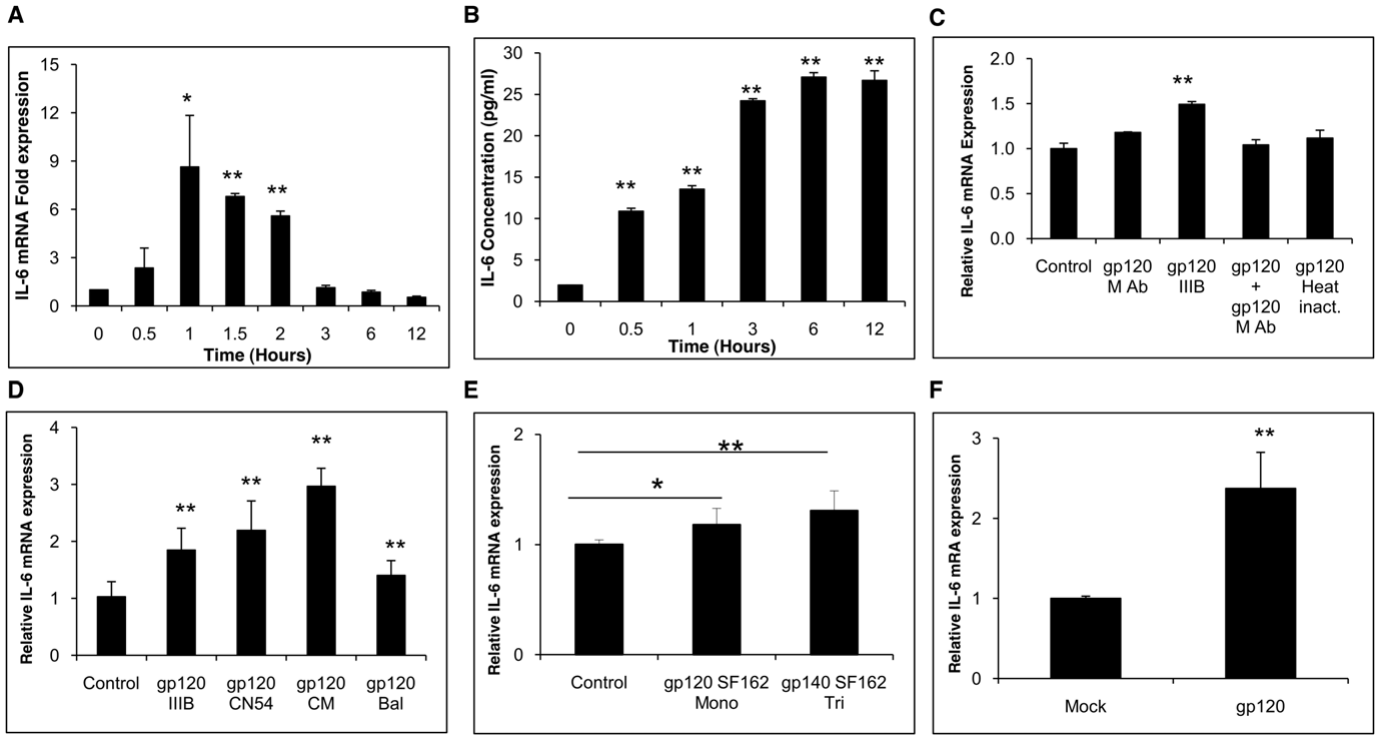
gp120 mediated increased levels of IL-6 in primary human fetal astrocytes. 1×10^6^ primary human fetal astrocytes from each of three different donors were treated with 20 nM gp120 IIIB for specific lengths of time and the cells were harvested to obtain mRNA. The cell culture supernatants were collected at the times indicated and used to quantify the level of IL-6 protein expression using a Bio-Plex assay. Levels of IL-6 mRNA expression as measured by real-time RT-PCR peaked at 1 hour (A) and IL-6 protein expressions peaked at 6 hours (B). Monoclonal antibody for gp120 was used to negate the gp120 mediated response and thus served as another control. Heat inactivated gp120 was compared with untreated control to show gp120 specific IL-6 expression (C). Different strains of gp120 (D) and monomeric gp120 SF162 and trimeric gp140 SF162 (E) increased the levels of IL-6 differentially. Primary astrocytes from different donors were transfected with gp120 plasmid in order to compare the levels of IL-6 expression after 2 hours (D). Each bar represents mean ± SE of 3 experiments with each experiment done in triplicates. The statistical significance was calculated using student's t test and * and ** denotes p value of ≤0.05 and ≤0.01, respectively.

To confirm the induction of IL-6 expression in response to gp120, human primary astrocytes obtained from 3 different donors were treated with 20 nM gp120IIIB for various lengths of time that ranged from 30 min to 12 hours, after which mRNA and cell culture supernatants were collected. Expression levels of IL-6 mRNA peaked at 1 hour (Figure 2A) followed by a gradual decline, while protein expression in the supernatants peaked at 6 hours (Figure 2B). All donors used in this study showed similar kinetic patterns with different levels of induction (6.3±0.2, 8.4±0.3, and 11.3±0.7 fold peak mRNA expression). In order to determine whether the observed response was specific for gp120, astrocytes were treated with heat-inactivated gp120. IL-6 expression was not significantly different in the treatment with heat-inactivated gp120 compared to the untreated control (Fig. 2C). We also treated astrocytes with a gp120 protein-antibody complex, and this further confirmed the specificity of gp120 (Figure 2C) as the gp120-immune complex did not induce IL-6 expression.

In addition to CXCR4, gp120 can also bind to CCR5 as a co-receptor. Astrocytes have been reported to express CCR5 in addition to CXCR4 on their surface [28], [29], [30]. In order to determine the differential effect of various strains of gp120 based on its tropism, primary astrocytes were treated with 20 nM of either gp120 CN54, gp120 CM or gp120 Bal. gp120 CN54, CM and Bal strains are M-tropic strains and all showed over expression of IL-6 (Figure 2D) at variable levels (2.2±0.21, 3±0.13 and 1.4±0.11 fold for gp120 CN54, CM and Bal respectively) . Previous studies have shown that the trimeric form of gp120 elicited a potent neutralizing response as compared to monomeric gp120 [31], thus providing a better immunogen for vaccination. In order to determine the inflammatory response of astrocytes to both of the forms of gp120, we used monomeric and trimeric forms of the gp120 and gp140 from SF162 strain. Both monomeric and trimeric forms increased the expression of IL-6 (1.2±0.1 and 1.3±0.1 fold respectively) (Figure 2E) to similar levels. Furthermore, in order to determine the effect of transfection on the primary astrocytes, a plasmid encoding gp120 was transfected using electroporation. The expression levels of IL-6 (2.38±0.44 fold) in the transfected cells were comparable to those observed when gp120 was added exogenously (Figure 2F).

### gp120 translocated NF-κB1 from cytoplasm to nucleus in astrocyte cells

**Figure 3:**
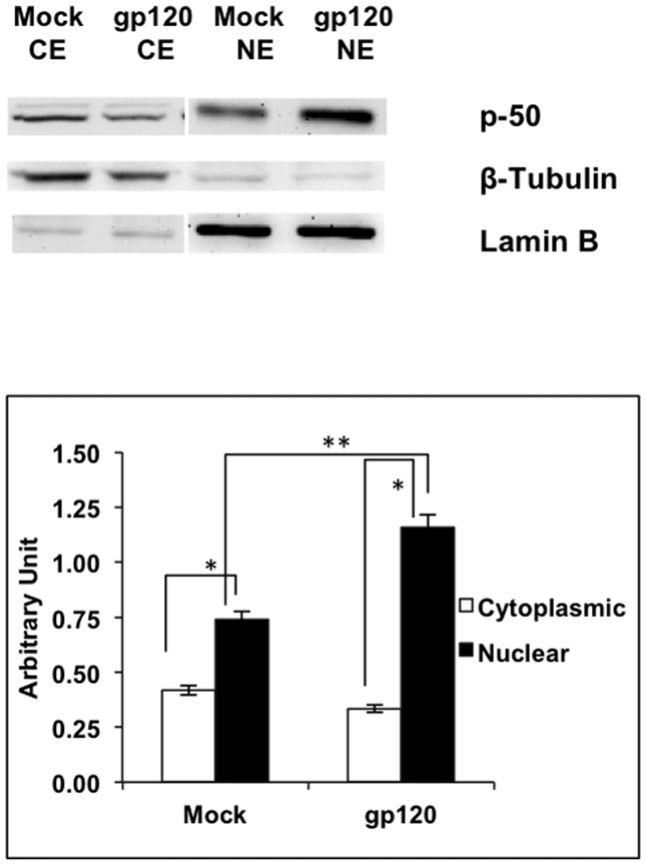
Increased NF-κB-translocation in gp120-transfected astrocytes. SVGA cells were either mock-transfected or were transfected with gp120 plasmid for a period of 6 hours followed by separation of cytosolic and nuclear fractions. These proteins were electrophoresed on 10% SDS gel and transferred to PVDF membrane. A representative western blot with lane-1 (mock-transfected SVGA cytosolic fraction), lane-2 (gp120-transfected SVGA cytosolic fraction), lane-3 (Mock-transfected SVGA nuclear fraction) and lane-4 (gp120-transfected SVGA nuclear fraction) is shown. The expression levels of p50 were normalized to their respective compartmental housekeeping genes (LaminB for nucleus and β-tubulin for cytoplasm) as loading controls. The bars, shown in the chart show normalized values of the band intensities for p50 over loading controls for appropriate compartments (LaminB for nucleus and β-tubulin for cytoplasm). The bar chart represents the means and SE of the ratios of p50 to the appropriate housekeeping gene (i.e. laminB for nuclear extracts and β-tubulin for cytoplasmic extracts). The means and SE values are from 3 independent experiments. The statistical significance was calculated using student's t test and * and ** denotes p value of ≤0.05 and ≤0.01, respectively.

We then wished to determine the signal transduction pathway involved in IL-6 induction. We examined the NF-κB pathway because its role in the induction of inflammatory cytokines, including IL-6, has been well documented [32], [33], [34]. Nuclear translocation of p50 and/or p65 (RelB) has been shown to be associated with NF-κB activation. SVGA cells were transfected with either the plasmid encoding gp120 or were mock-transfected. In comparing the cytoplasmic and nuclear fractions of the gp120-transfected and mock-transfected cells, we observed that gp120-transfected cells showed 2.74 fold higher p50 translocation as compared to mock-transfected cells (p<0.001) (Figure 3). In mock transfected cells there was 1.76±0.03 fold difference between nuclear and cytosolic fractions, (p<0.05), whereas in gp120 transfected cells the difference was 4.91±0.15 fold (p<0.001).

### gp120 activates NF-κB by inducing phosphorylation of IκBα in human fetal astrocytes

**Figure 4:**
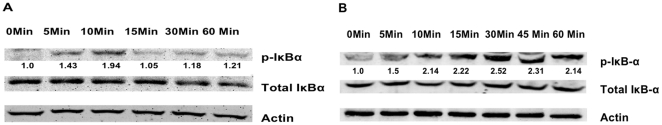
gp120 mediated increase in phosphorylation of IκB-α in primary human fetal astrocytes. Primary human astrocytes from two donors were treated with 20 nM gp120 IIIB, cells were collected and lysed using RIPA buffer at different times. These proteins were electrophoresed on 10% SDS gel and transferred to PVDF membrane. Antibodies against p-IκBα, total IκBα and β-actin were used for western blotting and membrane was read on densitometer. Values below the lanes show band intensities of the respective bands for both the donors (A & B). The increase in p-IκB-α was estimated by calculating the ratios of p-IκB-α to total IκB-α. Actin was used as loading control.

Inhibitory kappa B kinase is an enzyme that phosphorylates and releases IκBα from the p50/p65 heterodimer, thus yielding active NF-κB. Levels of p-IκB-α were measured to determine whether NF-κB activation plays a role in IL-6 induction. Primary astrocytes were treated with exogenous gp120 IIIB and the levels of phosphorylated IκB-α were measured in whole cell lysates. Although the peak levels were observed at different times for the two donors, IκB-α showed a time-dependent increase in phosphorylation as compared to total IκBα in astrocytes from both the donors (Figure 4A & 4B). Peak levels of phosphorylated IκB-α were observed at 10 min (1.94 fold higher) for donor-1 and 30 min (2.52 fold higher) for donor-2.

### Specific antagonists of NF-κB and siRNA targeted against NF-κB abrogated the gp120 mediated increase in IL-6 expression

**Figure 5:**
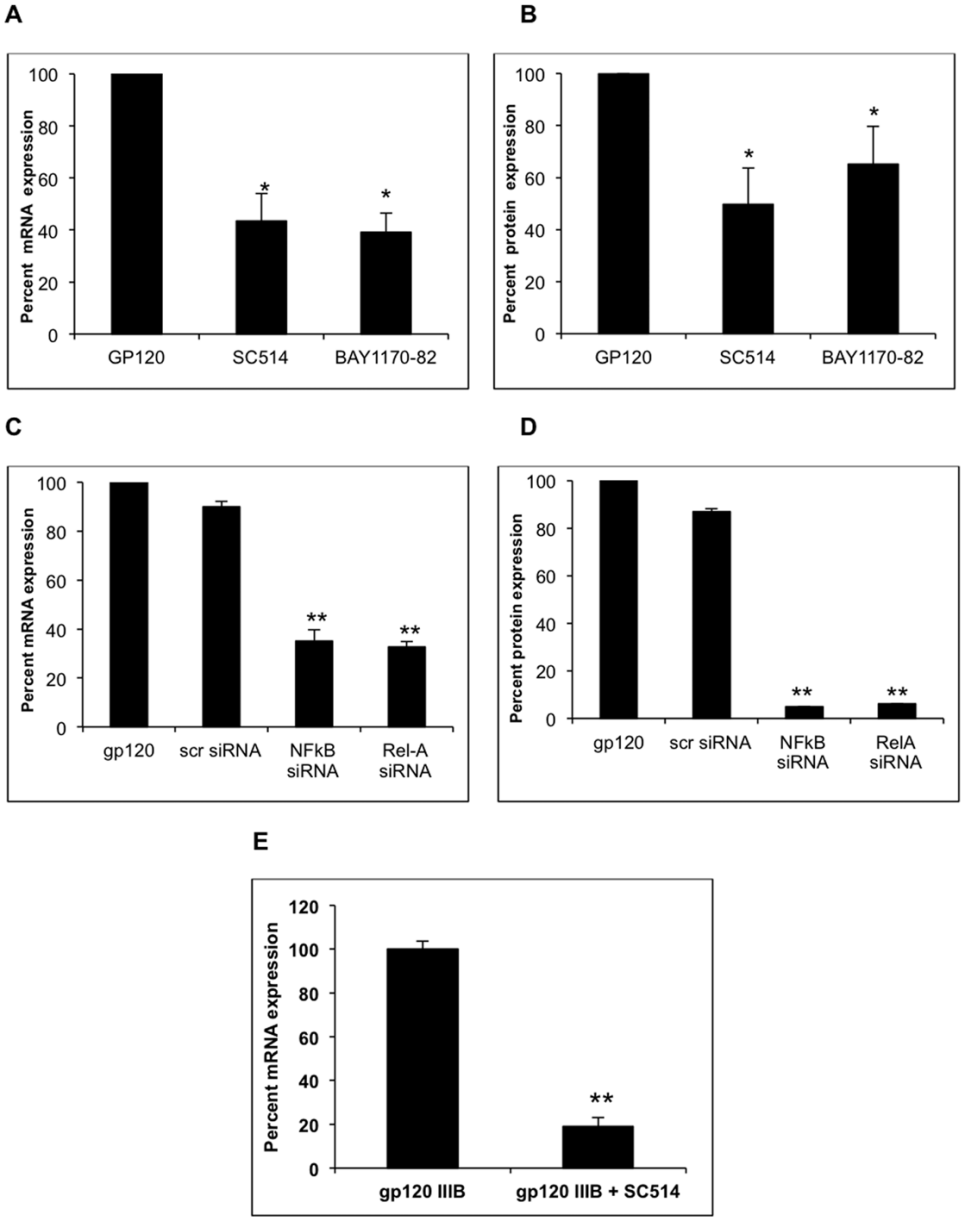
Inhibition of gp120-induced IL-6 expression by chemical inhibitors and siRNA specific for the NFκB pathway. SVGA astrocytes were treated with 10 µM SC514 (IKK-2 Inhibitor) and BAY11-7082 (IKKβ inhibitor) 24 hours prior to the transfection. 1×10^6^ SVGA astrocytes were transfected with 1 µg gp120 DNA in the presence of inhibitor, which was also maintained throughout experiments. IL-6 mRNA (A) and protein (B) was measured at 6 and 48 hours, post transfection respectively. For siRNA experiments, astrocytes were transfected with 50 nmoles of either scrambled, NFκB, or RelA siRNAs for 48 hours before gp120 transfection. IL-6 mRNA (C) and protein (D) was measured at 6 and 48 hours after gp120 transfection on cells previously transfected with siRNAs. (E) SVGA astrocytes were treated with 20 nM gp120IIIB. 10 µM SC514 was added to SVGA 1 hour prior to gp120 treatment. IL-6 mRNA was measured after 1 hour of gp120IIIB treatment. The mRNA is presented as relative percent mRNA expression with gp120 transfected cells as positive control. The protein concentration is presented as relative percent expression. Each bar represents mean ± SE of 3 experiments with each experiment done in triplicates. The statistical significance was calculated using student's t test and * and ** denotes p value of ≤0.05 and ≤0.01, respectively.

We hypothesized that the gp120-mediated IL-6 increase in astrocytes may occur through the NF-κB pathway as this pathway has been shown to be involved in IL-6 induction. We tested this hypothesis by using two chemical inhibitors and two unique siRNAs targeted against NF-κB. SC514 inhibits NF-κB activation by targeting IKK-2 [35], whereas BAY11-7082 blocks NF-κB activation by inhibiting the TNF-α-induced phosphorylation of IKKβ [36]. SVGA astrocytes were treated with 10 µM of either IKK-2 (SC514: IC50 = 14.5 µM) or IKKβ (BAY11-7082: IC50 = 11.2 µM) inhibitors for 24 hours prior to transfection with gp120 and maintained with inhibitors throughout the experiment. The concentration of the inhibitor was determined based on the IC50 as well as the viability of the cells, which were ∼90% viable at the inhibitor concentration that was used (data not shown). Expression levels of IL-6 mRNA were determined at 6 h after the termination of the transfection and IL-6 protein levels were measured at 48 h after the termination of the transfection. Both SC514 and BAY11-7082 successfully inhibited gp120-mediated expression of IL-6 mRNA by 56.5±10.5 percent and 60.8±7.3 percent, respectively (Figure 5A). Similarly, IL-6 protein levels were also reduced by 51.3±12.4% and 34.8±13.3%, respectively by SC514 and BAY11-7082 (Figure 5B).

In order to independently confirm the involvement of the NF-κB pathway in IL-6 induction we tested 2 siRNAs that were targeted against NF-κB. NF-κB and Rel-A specific siRNA were transfected into astrocytes 48 hours prior to gp120 transfection. IL-6 mRNA and protein levels were determined as described above. The results of these experiments are shown in Figure 5C and 5D, respectively. Both NF-κB and Rel-A specific siRNA blocked >60 and >90 percent mRNA and protein expression, respectively. These experiments confirm the results from the chemical inhibition experiments and demonstrate that the gp120-mediated increase in IL-6 expression is dependent on the NF-κB pathway. We also determined whether exogenous gp120 mediated IL-6 expression could be abrogated with NF-κB antagonists. Indeed, when pre-treated with SC514 (IKK-2 inhibitor), gp120 mediated IL-6 expression was reduced by 80±4.13% (Figure 5E).

### Small interfering RNA targeted against gp120 abrogated IL-6 expression

**Figure 6:**
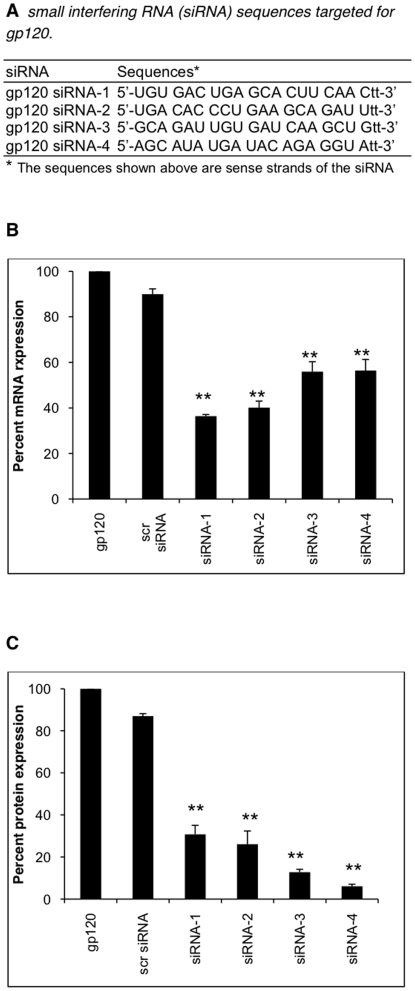
Inhibition of gp120-induced IL-6 expression by gp120-specific siRNA. Four gp120 siRNA (A) were designed using Ambion software and commercially synthesized by Ambion Inc. Only positive strand sequences are shown in the figure. Astrocytes were transfected with 50 nmoles each of scrambled or one of the 4 different siRNA for 48 hours before gp120 transfection. IL-6 mRNA (B) and protein (C) was measured at 6 and 48 hours, post-gp120 transfection. The mRNA is presented as relative percent mRNA expression with gp120 transfected cells as positive control. The protein concentration is presented as relative percent expression. Each bar represents mean ± SE of 3 experiments with each experiment done in triplicates. The statistical significance was calculated using student's t test and ** denotes p value of ≤0.01.

In this study we sought to address whether gp120 siRNA would block expression of IL-6. We designed 4 siRNA molecules (Figure 6A). These siRNA were commercially synthesized by Ambion Inc, Foster City, CA. The SVGA cells were transfected with different siRNA, 48 hour prior to gp120 transfection. Levels of IL-6 mRNA and protein expression were determined as described above. The siRNA sequences and results from these experiments are shown in Figure 5A. All 4 siRNAs inhibited IL-6 mRNA expression but the degree of inhibition was different with individual siRNAs. siRNA-1 was the most effective followed by siRNA-2, 3 and 4 in abrogating gp120-induced IL-6 RNA expression (Figure 6B). The efficacy of knockdown at the protein level was different with siRNA-4 blocking IL-6 protein expression most effectively (Figure 6C).

## Discussion

Multiple mechanisms have been proposed for HIV-1-induced neuroinflammation. In order to address the issue properly, it is critical to determine the potential mediators as well as the pathways involved in the induction or generation of such mediators. In the present study, gp120-mediated induction of IL-6 has been demonstrated to be dependent upon the NF-κB pathway. Interleukin-6 (IL-6) is a classical pro-inflammatory cytokine, which has been shown to be involved in response to various stimuli [37]. In our study using a human astrocyte cell line we observed that IL-6 mRNA expression reached a peak 6 h after transfection with a plasmid encoding gp120. In both types of cells IL-6 mRNA reached a peak at 1 h after the initiation of treatment with gp120 protein. These results are consistent with the previous studies by Ronaldson and Bendayan [16] who have found similar kinetics with respect to IL-1β, TNF-α and IL-6 after treatment of rat astrocytes with gp120. These results are also consistent with those obtained by Li et al, [2], who demonstrated that binding of HIV-1 to human fetal astrocytes resulted in increased production of IL-6. The authors confirmed the involvement of gp120 in mediating IL-6 over-expression by utilizing a truncated mutant virion, VSV-G pseudotype NL4-3, which was incapable of expressing gp120. The wild type VSV/NL4-3 was capable of inducing IL-6 in human fetal astrocytes, whereas the mutant failed to do so. In our experiments, as expected, there was a lag phase between the peak of mRNA and protein expressions and the results clearly demonstrated that gp120 increased the expression of IL-6 in SVGA astrocytes and in human primary astrocytes at the levels of both mRNA and protein. Our work demonstrates for the first time that gp120 induces IL-6 protein and mRNA in human astrocytes. In addition to CXCR4, astrocytes are reported to express CCR5 [28], [29], [30]. We showed differential expression levels of IL-6 in response to various strains of gp120. gp120IIIB was used as an X4 strain of gp120, while gp120 CN54, gp120 CM and gp120Bal were used as R5 stains. Our results clearly demonstrate that in addition to the gp120IIIB (X4 Strain), gp120 CN54, gp120CM and gp120Bal significantly altered IL-6 expression to various extents.

Using siRNA targeted against gp120, as well as transfections with empty vector, we have definitively shown that the increase in IL-6 observed is due to the presence of gp120. Interestingly, we observed differential regulation of IL-6 mRNA and protein expression after siRNA knockdown of gp120. Although gp120 siRNA 1 was the most effective siRNA in blocking expression of IL-6 mRNA, gp120 siRNA 4 was the most effective siRNA in blocking IL-6 expression at the level of protein. The reason for this discrepancy could be attributed to the fact that mRNA expression was monitored within 6 hrs after transfection whereas protein was monitored 48 hours after transfection and these siRNAs might have required more than 6 hour to exhibit full effect.

Nuclear Factor kappa B (NF-κB) has been shown to be involved in a wide array of cellular responses. The pathways in which NF-κB has been determined to be a key mediator include cell death, apoptosis and inflammation [32], [33], [34]. In rat astrocytes, induction of both MCP-1 and MCP-3 has been shown to be dependent upon NF-κB [38]. Ronaldson, et al. showed that pretreatment of astrocytes with a peptide inhibitor of NF-κB, SN-50 dramatically reduced the level of TNF-α elicited by gp120 treatment [29]. IL-6 production has also been found to be mediated though the NF-κB pathway in patients with rheumatoid arthritis [39], retinal microglia [40], lung pericytes [41] and mice splenocytes treated with LPS [42]. In our study, we sought to address whether NF-κB activation is involved in mediating gp120-induced IL-6 expression. We demonstrated that there is increased phosphorylation of IκBα along with significant translocation of p50 from the cytoplasm to the nucleus that is dependent upon gp120. This is similar to a report by Saha et al. [43] that demonstrates that gp120 activates the NF-κB pathway and leads to nuclear translocation of p50. Results obtained from pharmacological inhibitors and siRNA approach also confirmed that induction of IL-6 expression was dependent upon the NF-κB pathway. Taken together, the evidence presented in this paper along with results from other laboratories provides strong support for the involvement of NF-κB in mediating gp120 induction of IL-6. Along with its role in inducing inflammatory cytokines, NF-κB has also been demonstrated to be involved in regulating the responses to oxidative stress in astrocytes [44], [45]. Oxidative stress has been widely demonstrated as an important mechanism through which gp120 affects astrocytes [46]. Thus, activation of NF-κB by gp120 could be an important mechanism by which the cell protects itself from the oxidative stress associated with viral infection of the CNS.

Our data on the induction of IL-6 by gp120 are consistent with previously reported results. It should be noted that the results that we have observed using the two models of astrocyte exposure to gp120 (i.e. intracellular production of gp120 vs. extracellular administration of the protein) are somewhat different from each other in terms of kinetics and levels of IL-6 induction observed. These differences can be attributed to 2 differences in the models; one model utilizes extracellular exposure of primary astrocytes and the other model utilizes transfection of an immortalized cell line. We have utilized both models in order to determine the effects of gp120 on astrocytes regardless of the route of exposure. Our results not only confirm what other laboratories have reported with regard to extracellular exposure of astrocytes to gp120, but we have also shown that non-productive infection of astrocytes by HIV-1 may be a significant and persistent source of IL-6 in the CNS. It is important to note that the work presented here is the first demonstration that IL-6 is induced in human astrocytes in response to gp120. All previous reports in the literature have used either rat astrocytes or human mixed glial cultures.

This is an especially important finding because previously reported evidence suggests that IL-6 may be involved in the regulation of other cytokines such as TNF-α and IL-1β [25]. Taken together, this suggests that gp120-mediated activation of the NF-κB pathway may be critical therapeutic target for the treatment of HIV-related neuroinflammation. One approach that could be explored would be to try to interfere with the interaction between NF-κB and its binding site in the IL-6 promoter in astrocytes. Recent advances suggest that small RNAs or miRNA might be a useful tool for silencing promoters/enhancers [47], [48], [49], and such an approach might be applied to the IL-6 promoter in astrocytes. Also, recently Kim et al. have employed a novel approach to target specific cell types using antibody-based strategies [50]. Using their approach they were able to efficiently target delivery of siRNA specifically to T-cells via the CD67 receptor. Similarly, Wu et al. used microRNA delivery to astrocyte specific promoter as a tool for cancer therapy [51]. Such an approach might be further exploited in astrocytes by targeting these cells using GFAP to deliver siRNA/miRNA for NF-κB or IL-6 promotor. As successful antiretroviral treatment has extended the lifespan of those infected with HIV, the importance of finding novel treatments for the HIV-associated morbidities caused by chronic inflammation and oxidative stress, such as HAND, has become increasingly critical. Identification of a critical therapeutic target, as has been presented in this study, is an important step towards the development of more effective therapeutic regimens for neuroAIDS.

## Materials and Methods

### Cells and Reagents

All studies were reviewed and approved by Institutional Biosafety Committee and Institutional Review Board of UMKC. SVGA is a clone of a human fetal astrocyte cell line (SVG) [52] and was maintained in DMEM media supplemented with 10% FBS and 1% gentamicin at 37°C in 5% CO_2_ environment. Human fetal astrocytes were obtained from aborted fetal brain tissue and were grown in DMEM media supplemented with 10% FBS and 1% gentamicin at 37°C in 5% CO_2_ environment. The growing cells were >98% astrocytes as defined by GFAP staining (Data not shown). Lipofectamine™ 2000, and NF-κB inhibitors (IKK-2; SC514 and IKK-β; BAY1170-82) were obtained from Invitrogen Inc. (Carlsbad, CA) and Calbiochem (EMD Biosciences Inc., La Jolla, CA), respectively. The HIVgp120 plasmid, pSyn gp120 JR-FL (Catalog # 4598), recombinant HIV-1 IIIB gp120 (Catalog # 11784), HIV-1 CN54 gp120 (Catalog # 7749), recombinant HIV-1BaL gp120 (Catalog # 4961), HIV-1 gp120 Monoclonal (2G12) (Catalog # 1476) and HIV-1 gp120 CM (Catalog # 2968) were obtained from the NIH AIDS Research and Reference Reagent Program. Monomeric gp120 SF162 and trimeric forms of gp140 SF162 were prepared as discussed earlier [53], [54]. The heat-inactivated gp120 was prepared by heating gp120 at 65°C for 30 minutes. Negative control samples with gp120-immune complex treatments were also included. Monoclonal antibody gp120 (2G12) was mixed with gp120IIIB for 30 minutes in 10∶1 proportion to make immune complex prior to addition to the cells. Small interfering RNAs (siRNA) targeted against gp120 were designed using Ambion software and then synthesized by Ambion Inc. (Applied Biosystems, Foster City, CA). Pre-designed siRNA for NF-κB (P/N AM51331; id 5213) and Rel-A (P/N 4390824; id s11914) were also purchased from Ambion Inc. (Applied Biosystems). Specific antibodies for p50, lamin-B and β-tubulin were obtained from Santa Cruz Biotechnologies (Santa Cruz, CA).

### Transfection

The SVGA cells were transfected with Lipofectamine™ 2000 as recommended by the manufacturer. Briefly, 1×10^6^ cells were transiently transfected with 1 µg pSyn gp120 JR-FL for a period of 5 h in serum-free medium. The transfection was terminated after 5 h by the addition of complete media. The cells were harvested and total RNA was extracted using RNeasy kit from Qiagen (Valencia, CA). Cytokine expression was measured after 6, 12, 24, 48 and 72 h after the transfection was terminated. For NF-κB inhibition experiments, the cells were treated with 10 µM antagonists for 24 hours prior to the start of transfection. siRNA transfections were performed using Lipofectamine™ 2000 48 hrs prior to gp120 transfection. Briefly, 50 nmoles of siRNA was transfected into each well containing 1×10^6^ astrocytes in serum-free media. The transfection media was replaced after 24 hours with the fresh DMEM containing 10% FBS and the cells were incubated for 24 hours. The cells were then transiently transfected with gp120 and the cytokine levels were determined as described below. Controls in these experiments included mock transfection with equimolar empty plasmid and scrambled siRNA to compare siRNA trasfected cells.

### Electroporation of primary astrocytes for transfection of gp120

Transfections were performed as described in the manufacturer's protocol. Briefly, 8×0^6^ cells were mixed with the transfection reagent supplied with the kit (Amaxa rat astrocyte, VPI-1006) and the cells were placed in a cuvette. 5 µg of gp120 plasmid was added to the cells and electroporation was performed using a unique pulse program (T-020) for rat astrocyte cells which was optimized for human astrocytes. The cells were allowed to recover for 30 minutes with 500 µl DMEM media in the cuvette and further diluted with DMEM followed by 30 minute incubation. With the optimum conditions, the transfection efficiency was found to be 60–70% as measured with GFP expression (Data not shown) and the cell viability ranged from 45–60%. 2×10^6^ cells were plated per well.

### Real time RT-PCR and IL-6 protein assay

The cells were harvested and total RNA was extracted using RNeasy kit from Qiagen (Valencia, CA) at 6, 12, 24, 48 and 72 hours after the transfection was terminated. IL-6 mRNA was measured in Real-Time RT-PCR using forward primer (5′ GGT ACA TCC TCG ACG GCA TC 3′), reverse primer (5′ CCA GTG CCT CTT TGC TGC TT 3′), and probe (5′ FAM CAG CCC TGA GAA AGG AGA CAT GTA ACA GGA AA-3′ BHQ) in a Bio-Rad iCycler. The reaction conditions were as follows: reverse transcription at 50°C for 30 min, 95°C for 15 min and 50 cycles at 95°C for 15 sec and 57.5°C for 1 min. To normalize gene expression, HPRT was amplified in a separate reaction using the following primers and conditions: forward primer: 5′GCT TTC CTT GGT CAG GCA GTA 3′; reverse primer: 5′ CCA ACA CTT CGT GGR GTC CTT T 3′; reverse transcription at 50°C for 30 m, 95°C for 15 m and 45 cycles at 95°C for 15 sec and 55°C for 30 sec The data was analyzed using the equation 2^−ΔΔCT^ method as described previously [55]. Cell culture supernatants were collected at different times after transfection and IL-6 protein concentration was determined using a Bio-Plex System (Life Science Research, Hercules, CA). The protein expression was measured by comparing the values with the 5PL-standard curve using Bio-Plex Manager 5.0 software.

### Western blotting

SVGA cells were harvested at given time points and nuclear and cytoplasmic extracts were prepared using the NE-PER Nuclear extraction kit (Pierce, Rockford, IL) as per the manufacturer's directions. The cells were lysed with RIPA Buffer (Boston BioProducts, Ashland, MA), followed by homogenization for 15 sec and centrifugation at 12000 RPM for 5 minutes to eliminate cell debris. Protein concentrations were estimated using Pierce BCA protein assay (Pierce). 20 µg of protein was loaded on a 12% acrylamide gel and electrophoresed for at 90 V for 120 min and transferred to a PVDF membrane at 350 mA for 70 min. Phosphorylated-IκBα and total IκBα were detected using Phospho-IκB-α (Ser32) (14D4) (1∶1000) and IκB-α (#9242) (1∶1000) primary antibodies (Cell Signaling, Danvers, MA ), respectively. The band intensities were normalized using total IκBα. The expression of p50 was detected using NF-κB p50 (H-119) (1∶1000) primary antibody (Santa Cruz Biotechnology, Inc.) and expression was normalized using Lamin-B as an endogenous control for the nuclear extracts and β-tubulin as an endogenous control for the cytoplasmic extracts. Lamin B and β-tubulin were detected using Lamin B (C-20) (1∶1500) and β Tubulin (D-10) (1∶1500) primary antibodies respectively (Santa Cruz Biotechnology, Inc.). HRP conjugated secondary antibodies were used to detect the primary antibodies and detection of protein bands was performed using BM Chemiluminescence Western Blotting Substrate (POD) (Roche Applied Sciences, Indianapolis, IN). Quantification was done using spot densitometry with FluorChem HD2 software (Alpha Innotech, San Leandro, CA).

### Statistical analysis

Data are expressed in means ± SE of 3 experiments with each experiment done in triplicates. The statistical significance was calculated in student's t test and a p value<0.05 was considered significant.
